# Polycythemia Vera and Essential Thrombocythemia Patients Exhibit Unique Serum Metabolic Profiles Compared to Healthy Individuals and Secondary Thrombocytosis Patients

**DOI:** 10.3390/cancers13030482

**Published:** 2021-01-27

**Authors:** Nuria Gómez-Cebrián, Ayelén Rojas-Benedicto, Arturo Albors-Vaquer, Beatriz Bellosillo, Carlos Besses, Joaquín Martínez-López, Antonio Pineda-Lucena, Leonor Puchades-Carrasco

**Affiliations:** 1 Drug Discovery Unit, Instituto de Investigación Sanitaria La Fe, 46026 Valencia, Spain; nuria_gomez@iislafe.es (N.G.-C.); ayelen.rojas@externos.isciii.es (A.R.-B.); aralva@alumni.uv.es (A.A.-V.); apinedal@unav.es (A.P.-L.); 2 Department of Pathology, Hospital Del Mar Medical Research Institute, 08003 Barcelona, Spain; bbellosillo@parcdesalutmar.cat; 3 Department of Hematology, Hospital Del Mar Medical Research Institute, 08003 Barcelona, Spain; 92717@hospitaldelmar.cat; 4 Department of Hematology, Hospital Universitario 12 de Octubre, Universidad Complutense de Madrid, 28040 Madrid, Spain; jmarti01@med.ucm.es; 5 Molecular Therapeutics Program, Centro de Investigación Médica Aplicada, Universidad de Navarra, 28040 Pamplona, Spain

**Keywords:** myeloproliferative neoplasms, metabolomics, nuclear magnetic resonance, metabolic profile

## Abstract

**Simple Summary:**

Current diagnosis of myeloproliferative neoplasms (MPNs), including polycythemia vera (PV) and essential thrombocythemia (ET), is controversial due to limitations associated with the lack of reproducibility, subjectivity and the presence of common somatic mutations in the driver genes. Metabolomics represents a powerful approach to identify altered metabolites that can differentiate between disease status at the time of diagnosis. The objective of this study was to characterize the serum metabolic profile of MPNs patients (PV and ET) and compare it with healthy controls (HC) and secondary thrombocytosis (ST) patients. The analysis revealed metabolites following similar trends between PV and ET patients, as well as unique significant differences in the serum metabolite levels of MNPs patients compared to HC and ST patients. These results could contribute to better differentiate patients with these diseases from HC and ST patients.

**Abstract:**

Most common myeloproliferative neoplasms (MPNs) include polycythemia vera (PV) and essential thrombocythemia (ET). Accurate diagnosis of these disorders remains a clinical challenge due to the lack of specific clinical or molecular features in some patients enabling their discrimination. Metabolomics has been shown to be a powerful tool for the discrimination between different hematological diseases through the analysis of patients’ serum metabolic profiles. In this pilot study, the potential of NMR-based metabolomics to characterize the serum metabolic profile of MPNs patients (PV, ET), as well as its comparison with the metabolic profile of healthy controls (HC) and secondary thrombocytosis (ST) patients, was assessed. The metabolic profile of PV and ET patients, compared with HC, exhibited higher levels of lysine and decreased levels of acetoacetic acid, glutamate, polyunsaturated fatty acids (PUFAs), scyllo-inositol and 3-hydroxyisobutyrate. Furthermore, ET patients, compared with HC and ST patients, were characterized by decreased levels of formate, N-acetyl signals from glycoproteins (NAC) and phenylalanine, while the serum profile of PV patients, compared with HC, showed increased concentrations of lactate, isoleucine, creatine and glucose, as well as lower levels of choline-containing metabolites. The overall analysis revealed significant metabolic alterations mainly associated with energy metabolism, the TCA cycle, along with amino acid and lipid metabolism. These results underscore the potential of metabolomics for identifying metabolic alterations in the serum of MPNs patients that could contribute to improving the clinical management of these diseases.

## 1. Introduction

Philadelphia chromosome-negative (Ph-neg) myeloproliferative neoplasms (MPNs) are a group of blood disorders characterized by the clonal expansion of an abnormal hematopoietic progenitor cell in the bone marrow. Most common MPNs include polycythemia vera (PV), the most predominant MPNs condition accounting for 45% of all cases, and essential thrombocythemia (ET), which accounts for 25% of MPNs [1,2]. Clinically, PV is characterized by increased production of red blood cells (erythrocytosis) and may also be associated with elevated white cell (leukocytosis) and platelets (thrombocytosis) levels in blood [3,4], while ET is sustained by thrombocytosis and megakaryocytic proliferation [4,5]. Regarding molecular features, genetic mutations in three driver genes, including JAK2, CALR and myeloproliferative leukemia (MPL), can be found in both PV and ET patients [6,7].

Diagnosis of MPNs, currently performed according to the 2016 revised World Health Organization (WHO) classification of myeloid neoplasms [4], rely on a combination of clinical, morphological and molecular parameters, including the identification of driver mutations, such as JAK2V617F or CALR [8]. However, the somatic mutations in the driver genes are not exclusive of a particular MPN, and their absence does not preclude any of these neoplasms. While the presence of JAK2 mutation is expected in PV, approximately 90% of patients with ET express mutually exclusive JAK2, CALR, or MPL mutations [5]. Additionally, the diagnosis of ET patients can be hampered by their clinical resemblance with reactive or secondary thrombocytosis (ST) [9]. In this context, the precise discrimination at the time of diagnosis between PV and ET patients remains a major clinical challenge [8].

Metabolomics is defined as the comprehensive analysis of the metabolites present in a biological sample [10]. This approach has been shown to be a powerful tool for the detection of metabolic deregulations associated with diverse cancers, leading to the identification of specific sets of metabolites that discriminate between different disease status [11,12,13]. Particularly, metabolomics has recently enabled the discrimination between different hematological diseases through the analysis of patient serum metabolic profiles [11,14,15,16,17,18,19]. In this context, the characterization of the serum metabolic profile of MPNs patients could contribute to a better understanding of the molecular mechanisms underlying these diseases and could help to improve patient management. In this pilot study, the potential of NMR-based metabolomics to characterize the specific serum metabolic profile of MPNs patients (PV and ET) was assessed.

## 2. Results

### 2.1. Untargeted Analysis of Serum Metabolic Profile

**Figure 1:**
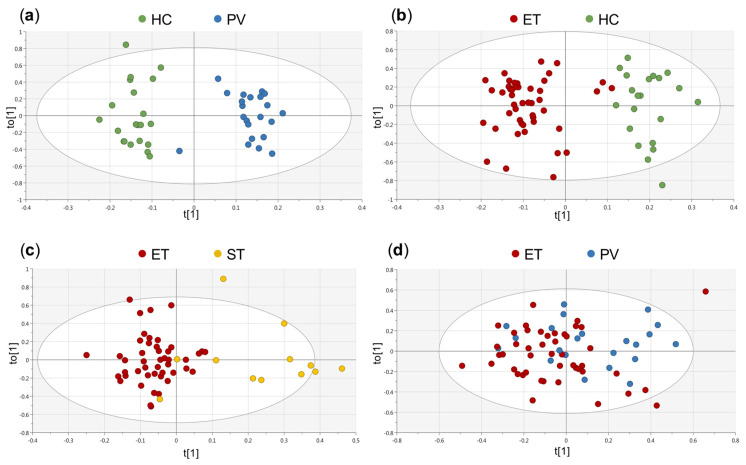
Orthogonal partial least squares discriminant analysis (OPLS-DA) scores showing the separation between the groups of study. (**a**) healthy controls (HC) (•) vs. polycythemia vera (PV) (•): R^2^Y = 0.909, Q^2^Y = 0.721, (**b**) HC (•) vs. essential thrombocythemia (ET) (•): R^2^Y = 0.82, Q^2^Y = 0.747, (**c**) ET (•) vs. secondary thrombocytosis (ST) (•): R^2^Y = 0.641, Q^2^Y = 0.463 and (**d**) PV (•) vs. ET (•): R^2^Y = 0.175, Q^2^Y = −0.0943. HC: healthy control, PV: polycythemia vera, ET: essential thrombocythemia, ST: secondary thrombocythemia.

Multivariate statistical analyses were based on principal component analysis (PCA) and orthogonal partial least square discriminant analysis (OPLS-DA). First, a non-supervised approach (i.e., PCA) was conducted to explore sample heterogeneity, and the potential impact of different clinical variables (group of study, age, gender, CALR and JAK2V617F mutational status) on the metabolic profiles of the individuals Figure S1. This analysis did not reveal significant sample clustering according to any of these variables. To further evaluate the differences between groups of study, OPLS-DA models were performed. Figure 1 shows the score plots generated for each comparison included in the study: HC vs. PV (R^2^Y = 0.909, Q^2^Y = 0.721), HC vs. ET (R^2^Y = 0.82, Q^2^Y = 0.747), ST vs. ET (R^2^Y = 0.641, Q^2^Y = 0.463) and PV vs. ET (R^2^Y = 0.175, Q^2^Y = −0.0943).

The first three models revealed significant differences between the metabolic profile of PV and ET patients, when compared with HC, and also between ET and ST patients. Nevertheless, the model generated for the comparison between PV and ET patients did not exhibit any predictive power (Q^2^Y = −0.0943). For all these OPLS-DA models, the most relevant variables correlated to the separation between groups of samples were extracted from the corresponding loading plots and the variable importance in projection (VIP) lists. Additionally, an OPLS-DA model for the comparison between HC and MPN (PV and ET) patients was generated (Figure S2, R^2^Y = 0.798, Q^2^Y = 0.701). The Q^2^Y value (predictive ability parameter, estimated by cross-validation) obtained for this model was comparable to the Q^2^Y values obtained when analyzing each MPN individually Figure 1. Moreover, when analyzing the NMR regions mostly contributing to this model, there was a significant overlap with the relevant regions identified in the analyses of the OPLS-DA models for the comparison of HC vs. ET and HC vs. PV, respectively. Therefore, to better examine the specific metabolic differences between the different groups of individuals included in the study, further analyses were based on the results obtained for each individual comparison.

Finally, a SUS-plot [20,21] for the comparison between HC vs. ET and HC vs. PV models was carried out to further explore the presence of shared (metabolites aligned with the diagonals) and/or unique (metabolites aligned with the axes) metabolic differences specific either to PV or ET patients when compared with HC Figure S3. In this plot, almost all NMR regions included in the OPLS-DA models were aligned with the diagonal, revealing a significant overlap of the most relevant NMR regions discriminating PV and ET patients from HC, respectively. This result is also in accordance with the fact that no significant metabolic differences were identified in the multivariate analysis for the discrimination of PV and ET patients.

### 2.2. GSEA of a Transcriptomic Study

Previous studies focused on the characterization of the transcriptomic profile of MPNs patients [15,22] did not specifically address the identification of changes in metabolic pathways. In this study, a specific GSEA, based on publicly available data from an MPNs transcriptomic study [15], focused on the identification of significantly altered metabolic pathways in PV and ET patients was carried out. The analysis revealed the existence of 7 metabolic pathways significantly deregulated (*p* adj < 0.05) when comparing PV and HC, and 3 when the comparison was carried out between ET and HC. However, no significant metabolic alterations were found in the comparison between PV and ET patients Table 1. Similar to the results obtained from the analysis of the serum metabolic profile, there was a significant overlap in the altered metabolic pathways when comparing PV and ET patients with HC. Particularly, the GSEA analysis revealed that the tricarboxylic acid (TCA) cycle and oxidative phosphorylation were the main metabolic pathways altered in these comparisons.

### 2.3. Univariate Analysis of Serum Metabolic Profiles

**Figure 2:**
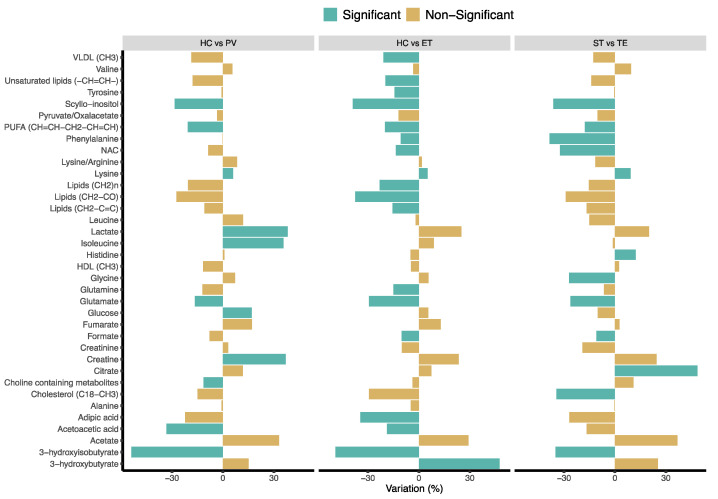
Variations in the level of the 36 metabolites identified by NMR in serum samples. The direction of the variation, considering the HC or ST groups as a reference. *p*-value calculated using the Mann–Whitney U test (*p* < 0.05 was considered significant). HC: healthy control, PV: polycythemia vera, ET: essential thrombocythemia, ST: secondary thrombocythemia. HDL: high-density lipoproteins, NAC: acetyl signals from glycoproteins, PUFAs: polyunsaturated fatty acids, VLDL: very-low-density lipoproteins.

To better examine the specific metabolic differences between the different groups of individuals included in the study, NMR regions contributing to the discrimination between the corresponding OPLS-DA models were analyzed. To that end, all NMR spectral regions corresponding to well-resolved signals were integrated for univariate analysis. A total of 51 NMR signals were integrated and assigned to 36 individual metabolites. Figure 2 summarizes the specific changes in the levels of these metabolites between the groups of study.

**Figure 3:**
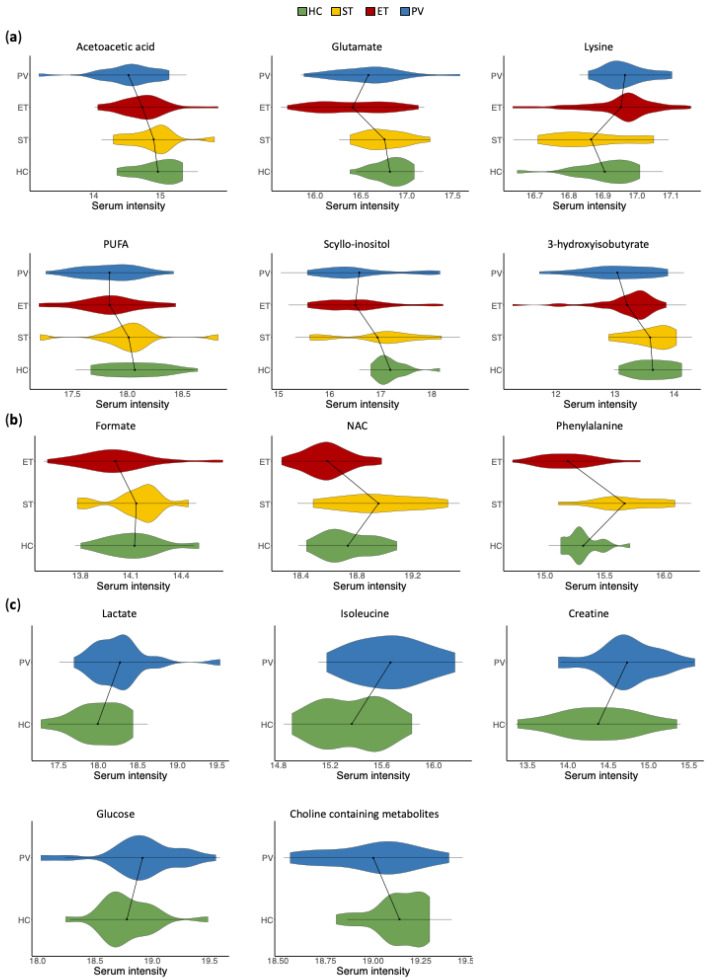
Violin plots representing the serum intensities (logarithmic scale) of statistically significant metabolic changes. Statistically significant alterations between the groups of study were observed in (**a**) metabolites that showed changes in both PV and ET patients, when compared with HC; (**b**) metabolites that were altered in ET (but not in PV) patients when compared with HC and ST; and (**c**) metabolites that were altered in PV (but not in ET) patients when compared with HC. HC: healthy control, PV: polycythemia vera, ET: essential thrombocythemia, ST: secondary thrombocythemia. HC: healthy control, PV: polycythemia vera, ET: essential thrombocythemia, ST: secondary thrombocythemia. HDL: high-density lipoproteins, NAC: acetyl signals from glycoproteins, PUFAs: polyunsaturated fatty acids, VLDL: very-low-density lipoproteins.

The analysis of the multivariate OPLS-DA models revealed that most of the metabolic changes observed in the comparisons between PV or ET patients with HC exhibited the same trend. However, some of these metabolic alterations were only statistically significant in one of the comparisons (PV vs. HC or ET vs. HC). The integrated analysis of all the metabolic changes facilitated the identification of three different sets of metabolites Figure 3a metabolites that exhibited statistically significant alterations in both PV and ET patients when compared with HC; Figure 3b metabolites that were significantly altered in only in ET (but not in PV) patients, when compared with HC and ST; and Figure 3c metabolites that were significantly altered only in PV (but not in ET) patients, when compared with HC.

Particularly, compared with HC, the metabolic profile of PV and ET patients exhibited higher levels of lysine and decreased levels of acetoacetic acid, glutamate, polyunsaturated fatty acids (PUFAs), scyllo-inositol and 3-hydroxyisobutyrate. Furthermore, ET patients, when compared with HC and ST patients, were characterized by decreased levels of formate, N-acetyl signals from glycoproteins (NAC) and phenylalanine, while the serum profile of PV patients, compared with HC, exhibited increased concentrations of lactate, isoleucine, creatine and glucose, as well as lower levels of choline-containing metabolites.

## 3. Discussion

The lack of specific clinical or molecular features in some PV and ET patients precludes their discrimination and hampers the accurate diagnosis of these disorders. To the best of our knowledge, this study represents the first global examination of the serum metabolic profile of PV and ET patients. To this end, NMR-based metabolomics was used to characterize the specific features of the serum metabolic profile of MPNs patients, as well as to evaluate the potential differences with those corresponding to HC and ST patients. Overall, the results suggested that PV and ET patients exhibit unique serum metabolic profiles associated with the molecular mechanisms responsible for these disorders.

The multivariate analysis of the metabolic profiles associated with MPNs patients, as well as those corresponding to HC and ST patients, revealed significant differences between these groups. Interestingly, no significant differences were found when comparing the serum profile of PV and ET patients. Similarly, statistically significant alterations in different metabolic pathways were found when comparing the transcriptomic profiles of PV or ET patients with HC, but no significant metabolic changes were identified when comparing PV and ET patients. These observations confirm the correlation between the findings of these two omics approaches. This could be explained by the clinical similarities between these two disorders.

Overall, the analysis of the multivariate statistical models revealed that most of the metabolic alterations observed in the comparisons between PV and ET patients with HC exhibited similar trends. These results are also in agreement with the results derived from the GSEA, based on changes in gene expression. Nevertheless, unique metabolic changes were also characterized. Thus, the comparison of ET patients with HC and ST patients revealed decreased levels of formate, NAC and phenylalanine. Furthermore, increased concentrations of lactate, isoleucine, creatine and glucose, as well as lower levels of choline-containing metabolites were identified in the serum profile of PV patients when compared with HC.

Some of the alterations identified in this study could reflect the high demands in energy and building blocks of fast proliferating cancer cells in MPN patients. These findings would be supported by the significant alterations in the TCA cycle and oxidative phosphorylation identified in the GSEA when comparing the transcriptomic profile of HC and PV or ET patients. Thus, serum metabolic alterations observed in PV and ET patients, such as changes in the levels of acetoacetic acid, 3-hydroxyisobutyrate and glutamate, could be associated with increased replenishment of metabolic intermediates for the TCA cycle. Specifically, acetoacetic acid, one of the most predominant ketone bodies, could be converted into acetyl-CoA, and 3-hydroxyisobutyrate transformed into succinyl-CoA. Glutamate is known to play several roles in cell proliferation [23], including its conversion to α-ketoglutarate for fueling the TCA. Finally, lower phenylalanine serum levels, observed in ET patients in our study, have been reported in cancer patients in previous studies [24], probably associated with its transformation into fumarate as anapleurotic substrate. Overall, enhanced degradation of these metabolites to fulfill highly activated energy metabolism in proliferating cancer cells may explain the lower levels observed in the serum profile of PV and ET patients. Finally, creatine levels, a key intermediate in energy metabolism that has been reported as elevated in different cancer patients [25], were found to be increased in PV and ET patients in this study, although this alteration was only statistically significant for PV patients.

Additionally, decreased serum levels of PUFAs in PV patients and choline-containing metabolites, observed in PV and ET patients in this study, could be associated with a higher demand for cell membrane synthesis. Lower serum PUFAs and choline levels have been previously reported and associated with increased risk in colorectal cancer patients [26,27]. Lower serum formate levels, observed in the serum profile of ET patients, have been previously reported in different cancer patients and associated with a higher proliferation of cancer cells [28]. Formate is a precursor of one-carbon (1C) units for nucleotide synthesis, a pathway that has been known to be altered in many cancers favoring cell proliferation [29].

Other metabolic alterations observed in PV patients in this study, such as higher lactate and isoleucine serum levels, could potentially be associated with other cancer-specific alterations. As it has been previously reported, cancer cell metabolism involves primarily the conversion of glucose ultimately to lactate following aerobic glycolysis, a phenomenon associated with the well-known Warburg effect [30]. Therefore, the elevated levels of lactate found in the serum of PV patients could be attributed to an increase in aerobic glycolysis. Elevated lactate levels have also been observed in acute myeloid leukemia [31] and other cancers [25] and have been associated with poor prognosis in hematological disorders [32]. Although this increase in lactate levels would be the result of increased glucose uptake by the tumor cells and diversion towards aerobic glycolysis [30], glucose levels were also found to be elevated in PV patients in this study. Serum glucose homeostasis involves a complex regulatory system [33]. Thus, additional metabolic alterations other than increased aerobic glycolysis rate could be affecting serum glucose homeostasis in these patients [34]. Finally, increased serum levels of isoleucine in PV patients, a branched-chain amino acid (BCAA) observed in this study, are in agreement with previous findings that have been reported in other cancer patients [11,25,35]. In these patients, increased protein turnover and muscle breakdown, associated with cancer cachexia, together with lower BCAAs consumption by tumor cells, may lead to high BCAAs serum levels in cancer patients.

## 4. Materials and Methods

### 4.1. Study Cohort

Serum samples from 101 individuals were collected and classified into HC (*n* = 21), PV (*n* = 22), ET (*n* = 46) and ST (*n* = 12) groups. Patients’ diagnosis was based on the WHO classification of Myeloid Neoplasms 2016 [4]. Samples included in the study were collected from patients after diagnosis and before treatment. Clinical characteristics of the individuals included in the study are summarized in Table 2. Further clinical details of the MPN patients included in the study are included in Table S1.

### 4.2. Serum Sample Collection

Patients’ recruitment was performed at the Hospital Universitario 12 de Octubre (Madrid, Spain) and the Hospital del Mar (Barcelona, Spain). Recruitment and sampling procedures were carried out in accordance with the Declaration of Helsinki and applicable local regulatory requirements and laws and after approval from the Ethics Committees of all participant institutions. Serum samples were processed following metabolomics specific standard operating procedures (SOPs) [11,14,36,37,38,39]. Samples were frozen and stored at −80 °C until analyzed using NMR spectroscopy.

### 4.3. NMR Sample Preparation

At the time of NMR analysis, serum samples were thawed on ice. Then, 300 µL of serum were added to 300 µL of buffer (140 mM Na_2_HPO_4_, 5 mM trimethylsilylpropionic acid-d4 sodium salt (TSP), 0.04% NaN_3_, pH 7.4, in 10% D_2_O). Finally, 550 µL of each sample was transferred to a 5 mm NMR tube for analysis.

### 4.4. NMR Sample Measurements

NMR measurements were acquired using a Bruker Avance II 600 MHz spectrometer equipped with a triple resonance cryoprobe with a cooled 13 C preamplifier (TCI) at an acquisition temperature of 310 K. NMR data acquisition was performed as previously described [11,13,39,40]. Briefly, standard Carr–Purcell–Meiboom–Gill (CPMG) [41] a spin–echo pulse sequence was collected for each sample. A total of 64 free induction decays (FIDs) collected into 74 K data points were used for the acquisition of the NMR spectra over a spectral width of 20 ppm and a relaxation delay of 4 s between FIDs. A presaturation water pulse of 25 Hz was applied throughout the relaxation delays to improve solvent suppression [41,42].

### 4.5. NMR Data Processing

After the acquisition, serum spectra were multiplied by a line-broadening factor of 1 Hz, and Fourier transformed. All spectra were automatically phased, baseline corrected and referenced to the methyl group signal of alanine (δ 1.47 ppm) using TopSpin 3.5 (Bruker BioSpin, Rheinstetten, Germany). Then, serum spectra were binned using Amix 3.9.15 (Bruker BioSpin) into 0.01 ppm wide rectangular buckets over the spectral region δ 8.60–0.50 ppm. The residual water (δ 5.10–4.36 ppm) and urea (δ 5.82–5.65) signal regions were excluded from the analysis to avoid interferences arising from differences in water suppression and variability from urea signal, respectively.

### 4.6. Multivariate Statistical Analysis

Bucket tables were imported into SIMCA-P 14.0 software (Umetrics AB, Umeå, Sweden). Data were Pareto scaled by dividing each variable by the square root of 1/SD, where SD represents the standard deviation value of each variable [43]. Then, principal component analysis (PCA) and orthogonal partial least squares discriminant analysis (OPLS-DA) were conducted. The default method of 7-fold internal cross-validation of the software was applied, from which Q^2^Y (predictive ability parameter, estimated by cross-validation) and R^2^Y (goodness of fit parameter) values were extracted. These statistical parameters were used for the evaluation of the quality of the OPLS-DA models obtained. Finally, shared and unique structures (SUS)—the plot was used for the evaluation of mutual and exclusive differences when comparing OPLS-DA models.

### 4.7. Transcriptomic Analysis

Raw data from the GSE61629 [15] study was obtained from the Gene Expression Omnibus (GEO; https://www.ncbi.nlm.nih.gov/geo) database [44,45]. All statistical analyses were conducted using the R 3.6.0 version [46].

Gene expression values were normalized using the “RMA” function form the “affy” R package [47]. Differential gene expression analysis between groups of samples was assessed using the “limma” R package [48]. Differential expression *p*-values were adjusted based on the Benjamini and Hochberg (BH) method [49]. Gene set enrichment analysis (GSEA) was conducted using functions implemented in the “mdgsa” R package [50]. The metabolic pathways defined by the Kyoto Encyclopedia of Genes and Genomes (KEGG) database [51,52,53] were used for the functional enrichment. Finally, metabolic pathways showing a Benjamini–Yekutieli (BY) [54] adjusted *p*-value (*p* adj) < 0.05 were defined as significantly deregulated.

### 4.8. Metabolite Quantification

Metabolite signals in the NMR spectra were assigned using the Bruker NMR Metabolic profiling database BBIOREFCODE 2.0.0 (Bruker BioSpin), in combination with other publicly available databases [55,56]. Both singlets and multiplets of the same metabolite were used, when possible, for identification and integration. Spectra were baseline corrected, aligned, and metabolite signals were integrated and quantified using NMRProcFlow v.1.2.28 [57]. The statistical significance of the differences between the means of the groups of the study was assessed using the Mann–Whitney U test. A *p*-value < 0.05 was considered statistically significant.

## 5. Conclusions

Overall, the results obtained in this pilot study suggest that the characterization of the serum metabolic profile of MPNs patients could provide useful information to better understand the molecular mechanisms underlying these diseases. Although further validation of the results, using complementary experimental approaches and larger independent patients’ cohorts would be necessary, the metabolomic analysis of serum samples from HC and MPNs patients revealed significant metabolic differences that could be used to differentiate between individuals with and without the disease.

## Figures and Tables

**Table 1:** Significantly enriched metabolic pathways identified in the gene set enrichment analysis (GSEA) for the GSE61629 transcriptomic study.

Comparison	Pathway Name	KEGG ID	LOR	*p*-Value	BY Adjusted
**ET vs. HC**	Oxidative phosphorylation	hsa00190	−0.4946	7.57 × 10^−8^	3.24 × 10^−5^
	Citrate cycle (TCA cycle)	hsa00020	−0.6648	1.87 × 10^−4^	4.00 × 10^−2^
	alpha-linolenic acid metabolism	hsa00592	0.7378	3.18 × 10^−4^	4.53 × 10^−2^
**PV vs. HC**	Citrate cycle (TCA cycle)	hsa00020	−0.9519	1.85 × 10^−8^	7.88 × 10^−6^
	Oxidative phosphorylation	hsa00190	−0.4006	1.14 × 10^−5^	1.76 × 10^−3^
	Carbon metabolism	hsa01200	−0.4138	1.24 × 10^−5^	1.76 × 10^−3^
	Valine, leucine and isoleucine degradation	hsa00280	−0.6365	2.36 × 10^−5^	2.52 × 10^−3^
	Starch and sucrose metabolism	hsa00500	0.6765	1.05 × 10^−4^	8.95 × 10^−3^
	Propanoate metabolism	hsa00640	−0.6678	1.48 × 10^−4^	1.06 × 10^−2^
	Pyruvate metabolism	hsa00620	−0.5943	4.15 × 10^−4^	2.53 × 10^−2^
**PV vs. ET**	−	−	−	−	−

BY: Benjamini–Yekutieli, ET: essential thrombocythemia, HC: healthy control, PV: polycythemia vera, ST: secondary thrombocythemia, TCA cycle: tricarboxylic acid cycle.

**Table 2:** Clinical characteristics of the patients included in the study.

Group	*n* (%)	Age (Mean ± SD)	Sex (m/f)	JAK2^V617^ (Mut/WT)	CALR (Mut/WT/NA)
HC	21 (20.8%)	58 ± 5.78	12/9	−/21/−	−/21/−
PV	22 (21.8%)	68.86 ± 14.57	14/8	22/−/−	−/1/21
ET	46 (45.5%)	61.89 ± 16.53	18/28	24/22	9/23/14
ST	12 (11.9%	60.17 ± 16.07	4/8	−/12/−	−/12/−

ET: essential thrombocythemia, f: female, HC: healthy control, m: male, Mut: mutant, *n*: sample size, NA: not available, PV: polycythemia vera, SD: standard deviation, ST: secondary thrombocythemia, WT: wild type.

## Data Availability

Transcriptomic data presented in this study are openly available in the Gene Expression Omnibus database (GEO; https://www.ncbi.nlm.nih.gov/geo) at GSE61629. Raw NMR spectra data (raw metabolomics data) analyzed in this study are available on request from the corresponding author.

## Supplementary Materials

The following information is available online at https://www.mdpi.com/2072-6694/13/3/482/s1. Table S1: Clinical details available for the MPN patients included in the study; Figure S1: principal component analysis (PCA) score plots corresponding to the samples included in the study; Figure S2: OPLS-DA scores plot for the comparison between HC vs. ET + PV; Figure S3: SUS-plot analysis correlating the OPLS-DA models of HC vs. PV (*x*-axis) and HC vs. ET (*y*-axis).

## References

[1] Rollison D.E., Howlader N., Smith M.T., Strom S.S., Merritt W.D., Ries L.A., Edwards B.K., List A.F. (2008). Epidemiology of Myelodysplastic Syndromes and Chronic Myeloproliferative Disorders in the United States, 2001–2004, Using Data from the NAACCR and SEER Programs. Blood.

[2] Moulard O., Mehta J., Fryzek J., Olivares R., Iqbal U., Mesa R.A. (2014). Epidemiology of Myelofibrosis, Essential Thrombocythemia, and Polycythemia Vera in the European Union. Eur. J. Haematol..

[3] Tonkin J., Francis Y., Pattinson A., Peters T., Taylor M., Thompson R., Wallis L. (2012). Myeloproliferative Neoplasms: Diagnosis, Management and Treatment. Nurs. Stand..

[4] Arber D.A., Orazi A., Hasserjian R., Thiele J., Borowitz M.J., Le Beau M.M., Bloomfield C.D., Cazzola M., Vardiman J.W. (2016). The 2016 Revision to the World Health Organization Classification of Myeloid Neoplasms and Acute Leukemia. Blood.

[5] Tefferi A., Barbui T. (2019). Polycythemia Vera and Essential Thrombocythemia: 2019 Update on Diagnosis, Risk-Stratification and Management. Am. J. Hematol..

[6] Nangalia J., Green T.R. (2014). The Evolving Genomic Landscape of Myeloproliferative Neoplasms. Hematol. Am. Soc. Hematol. Educ. Program..

[7] Fowlkes S., Murray C., Fulford A., De Gelder T., Siddiq N. (2018). Myeloproliferative Neoplasms (MPNs)—Part 1: An Overview of the Diagnosis and Treatment of the “Classical” MPNs. Can. Oncol. Nurs. J..

[8] Barbui T., Thiele J., Gisslinger H., Kvasnicka H.M., Vannucchi A.M., Guglielmelli P., Orazi A., Tefferi A. (2018). The 2016 WHO Classification and Diagnostic Criteria for Myeloproliferative Neoplasms: Document Summary and in-Depth Discussion. Blood Cancer J..

[9] Kutti J., Wadenvik H. (1996). Diagnostic and Differential Criteria of Essential Thrombocythemia and Reactive Thrombocytosis. Leuk. Lymphoma.

[10] Lindon J.C., Nicholson J.K., Holmes E., Everett J.R. (2000). Metabonomics: Metabolic Processes Studied by NMR Spectroscopy of Biofluids. Concepts Magn. Reson..

[11] Puchades-Carrasco L., Lecumberri R., Martínez-López J., Lahuerta J.-J., Mateos M.-V., Prósper F., San-Miguel J.F., Pineda-Lucena A. (2013). Multiple Myeloma Patients Have a Specific Serum Metabolomic Profile That Changes after Achieving Complete Remission. Clin. Cancer Res..

[12] Puchades-Carrasco L., Jantus-Lewintre E., Pérez-Rambla C., García-García F., Lucas R., Calabuig S., Blasco A., Dopazo J., Camps C., Pineda-Lucena A. (2016). Serum Metabolomic Profiling Facilitates the Non-Invasive Identification of Metabolic Biomarkers Associated with the Onset and Progression of Non-Small Cell Lung Cancer. Oncotarget.

[13] Gómez-Cebrián N., García-Flores M., Rubio-Briones J., López-Guerrero J.A., Pineda-Lucena A., Puchades-Carrasco L. (2020). Targeted Metabolomics Analyses Reveal Specific Metabolic Alterations in High-Grade Prostate Cancer Patients. J. Proteome Res..

[14] MacIntyre D.A., Jiménez B., Lewintre E.J., Martín C.R., Schäfer H., Ballesteros C.G., Mayans J.R., Spraul M., García-Conde J., Pineda-Lucena A. (2010). Serum Metabolome Analysis by 1H-NMR Reveals Differences between Chronic Lymphocytic Leukaemia Molecular Subgroups. Leukemia.

[15] Hasselbalch H.C., Thomassen M., Hasselbalch Riley C., Kjær L., Stauffer Larsen T., Jensen M.K., Bjerrum O.W., Kruse T.A., Skov V. (2014). Whole Blood Transcriptional Profiling Reveals Deregulation of Oxidative and Antioxidative Defence Genes in Myelofibrosis and Related Neoplasms. Potential Implications of Downregulation of Nrf2 for Genomic Instability and Disease Progression. PLoS ONE.

[16] Allegra A., Innao V., Gerace D., Bianco O., Musolino C. (2016). The Metabolomic Signature of Hematologic Malignancies. Leuk. Res..

[17] Steiner N., Müller U., Hajek R., Sevcikova S., Borjan B., Jöhrer K., Göbel G., Pircher A., Gunsilius E. (2018). The Metabolomic Plasma Profile of Myeloma Patients Is Considerably Different from Healthy Subjects and Reveals Potential New Therapeutic Targets. PLoS ONE.

[18] Du H., Wang L., Liu B., Wang J., Su H., Zhang T., Huang Z. (2018). Analysis of the Metabolic Characteristics of Serum Samples in Patients With Multiple Myeloma. Front. Pharmacol..

[19] Barberini L., Noto A., Fattuoni C., Satta G., Zucca M., Cabras M.G., Mura E., Cocco P. (2019). The Metabolomic Profile of Lymphoma Subtypes: A Pilot Study. Molecules.

[20] Wiklund S., Johansson E., Sjöström L., Mellerowicz E.J., Edlund U., Shockcor J.P., Gottfries J., Moritz T., Trygg J. (2008). Visualization of GC/TOF-MS-Based Metabolomics Data for Identification of Biochemically Interesting Compounds Using OPLS Class Models. Anal. Chem..

[21] Jiang L., Si Z.-H., Li M.-H., Zhao H., Fu Y.-H., Xing Y.-X., Hong W., Ruan L.-Y., Li P.-M., Wang J.-S. (2017). 1H NMR-Based Metabolomics Study of Liver Damage Induced by Ginkgolic Acid (15:1) in Mice. J. Pharm. Biomed. Anal..

[22] Skov V., Burton M., Thomassen M., Stauffer Larsen T., Riley C.H., Brinch Madelung A., Kjær L., Bondo H., Stamp I., Ehinger M. (2016). A 7-Gene Signature Depicts the Biochemical Profile of Early Prefibrotic Myelofibrosis. PLoS ONE.

[23] Cluntun A.A., Lukey M.J., Cerione R.A., Locasale J.W. (2017). Glutamine Metabolism in Cancer: Understanding the Heterogeneity. Trends Cancer.

[24] Miyagi Y., Higashiyama M., Gochi A., Akaike M., Ishikawa T., Miura T., Saruki N., Bando E., Kimura H., Imamura F. (2011). Plasma Free Amino Acid Profiling of Five Types of Cancer Patients and Its Application for Early Detection. PLoS ONE.

[25] Zhang X., Xu L., Shen J., Cao B., Cheng T., Zhao T., Liu X., Zhang H. (2013). Metabolic Signatures of Esophageal Cancer: NMR-Based Metabolomics and UHPLC-Based Focused Metabolomics of Blood Serum. Biochim. Biophys. Acta Mol. Basis Dis..

[26] Mika A., Kobiela J., Pakiet A., Czumaj A., Sokołowska E., Makarewicz W., Chmielewski M., Stepnowski P., Marino-Gammazza A., Sledzinski T. (2020). Preferential Uptake of Polyunsaturated Fatty Acids by Colorectal Cancer Cells. Sci. Rep..

[27] Nitter M., Norgård B., de Vogel S., Eussen S.J.P.M., Meyer K., Ulvik A., Ueland P.M., Nygård O., Vollset S.E., Bjørge T. (2014). Plasma Methionine, Choline, Betaine, and Dimethylglycine in Relation to Colorectal Cancer Risk in the European Prospective Investigation into Cancer and Nutrition (EPIC). Ann. Oncol..

[28] Pietzke M., Arroyo S.F., Sumpton D., Mackay G.M., Martin-Castillo B., Camps J., Joven J., Menendez J.A., Vazquez A. (2019). Stratification of Cancer and Diabetes Based on Circulating Levels of Formate and Glucose. Cancer Metab..

[29] Villa E., Ali E., Sahu U., Ben-Sahra I. (2019). Cancer Cells Tune the Signaling Pathways to Empower de Novo Synthesis of Nucleotides. Cancers.

[30] Warburg O. (1956). On the Origin of Cancer Cells. Science.

[31] Wojtowicz W., Chachaj A., Olczak A., Ząbek A., Piątkowska E., Rybka J., Butrym A., Biedroń M., Mazur G., Wróbel T. (2018). Serum NMR Metabolomics to Differentiate Haematologic Malignancies. Oncotarget.

[32] Sillos E.M., Shenep J.L., Burghen G.A., Pui C.-H., Behm F.G., Sandlund J.T. (2001). Lactic Acidosis: A Metabolic Complication of Hematologic Malignancies. Cancers.

[33] Matschinsky F.M., Wilson D.F. (2019). The Central Role of Glucokinase in Glucose Homeostasis: A Perspective 50 Years After Demonstrating the Presence of the Enzyme in Islets of Langerhans. Front. Physiol..

[34] Guo X., Li H., Xu H., Woo S., Dong H., Lu F., Lange A.J., Wu C. (2012). Glycolysis in the Control of Blood Glucose Homeostasis. Acta Pharma. Sin. B.

[35] Mayers J.R., Wu C., Clish C.B., Kraft P., Torrence M.E., Fiske B.P., Yuan C., Bao Y., Townsend M.K., Tworoger S.S. (2014). Elevation of Circulating Branched-Chain Amino Acids Is an Early Event in Human Pancreatic Adenocarcinoma Development. Nat. Med..

[36] Beckonert O., Keun H.C., Ebbels T.M.D., Bundy J., Holmes E., Lindon J.C., Nicholson J.K. (2007). Metabolic Profiling, Metabolomic and Metabonomic Procedures for NMR Spectroscopy of Urine, Plasma, Serum and Tissue Extracts. Nat. Protoc..

[37] Ghini V., Quaglio D., Luchinat C., Turano P. (2019). NMR for Sample Quality Assessment in Metabolomics. New Biotechnol..

[38] Dona A.C., Jiménez B., Schäfer H., Humpfer E., Spraul M., Lewis M.R., Pearce J.T.M., Holmes E., Lindon J.C., Nicholson J.K. (2014). Precision High-Throughput Proton NMR Spectroscopy of Human Urine, Serum, and Plasma for Large-Scale Metabolic Phenotyping. Anal. Chem..

[39] Vicente-Muñoz S., Morcillo I., Puchades-Carrasco L., Payá V., Pellicer A., Pineda-Lucena A. (2016). Pathophysiologic Processes Have an Impact on the Plasma Metabolomic Signature of Endometriosis Patients. Fertil. Steril..

[40] Pérez-Rambla C., Puchades-Carrasco L., García-Flores M., Rubio-Briones J., López-Guerrero J.A., Pineda-Lucena A. (2017). Non-Invasive Urinary Metabolomic Profiling Discriminates Prostate Cancer from Benign Prostatic Hyperplasia. Metabolomics.

[41] Meiboom S., Gill D. (1958). Modified Spin-Echo Method for Measuring Nuclear Relaxation Times. Rev. Sci. Instrum..

[42] Nicholson J.K., Foxall P.J., Spraul M., Farrant R.D., Lindon J.C. (1995). 750 MHz 1H and 1H-13C NMR Spectroscopy of Human Blood Plasma. Anal. Chem..

[43] Worley B., Powers R. (2013). Multivariate Analysis in Metabolomics. Curr. Metab..

[44] Edgar R., Domrachev M., Lash A.E. (2002). Gene Expression Omnibus: NCBI Gene Expression and Hybridization Array Data Repository. Nucleic Acids Res..

[45] Barrett T., Wilhite S.E., Ledoux P., Evangelista C., Kim I.F., Tomashevsky M., Marshall K.A., Phillippy K.H., Sherman P.M., Holko M. (2013). NCBI GEO: Archive for Functional Genomics Data Sets—Update. Nucleic Acids Res..

[46] R Core Team R: A Language and Environment for Statistical Computing. https://www.R-project.org.

[47] Gautier L., Cope L., Bolstad B.M., Irizarry R.A. (2004). Affy—Analysis of Affymetrix GeneChip Data at the Probe Level. Bioinformatics.

[48] Ritchie M.E., Phipson B., Wu D., Hu Y., Law C.W., Shi W., Smyth G.K. (2015). Limma Powers Differential Expression Analyses for RNA-Sequencing and Microarray Studies. Nucleic Acids Res..

[49] Benjamini Y., Hochberg Y. (1995). Controlling the False Discovery Rate: A Practical and Powerful Approach to Multiple Testing. J. R Stat. Soc. Ser. B Methodol..

[50] Montaner D., Dopazo J. (2010). Multidimensional Gene Set Analysis of Genomic Data. PLoS ONE.

[51] Kanehisa M. (2000). KEGG: Kyoto Encyclopedia of Genes and Genomes. Nucleic Acids Res..

[52] Kanehisa M., Sato Y., Furumichi M., Morishima K., Tanabe M. (2019). New Approach for Understanding Genome Variations in KEGG. Nucleic Acids Res..

[53] Kanehisa M. (2019). Toward Understanding the Origin and Evolution of Cellular Organisms. Protein Sci..

[54] Yekutieli D., Benjamini Y. (2001). The Control of the False Discovery Rate in Multiple Testing under Dependency. Ann. Statist..

[55] Wishart D.S., Feunang Y.D., Marcu A., Guo A.C., Liang K., Vázquez-Fresno R., Sajed T., Johnson D., Li C., Karu N. (2018). HMDB 4.0: The Human Metabolome Database for 2018. Nucleic Acids Res..

[56] Markley J.L., Ulrich E.L., Berman H.M., Henrick K., Nakamura H., Akutsu H. (2008). BioMagResBank (BMRB) as a Partner in the Worldwide Protein Data Bank (WwPDB): New Policies Affecting Biomolecular NMR Depositions. J. Biomol. NMR.

[57] Jacob D., Deborde C., Lefebvre M., Maucourt M., Moing A. (2017). NMRProcFlow: A Graphical and Interactive Tool Dedicated to 1D Spectra Processing for NMR-Based Metabolomics. Metabolomics.

