# Supplementary material for: Polycythemia Vera and Essential Thrombocythemia Patients Exhibit Unique Serum Metabolic Profiles Compared to Healthy Individuals and Secondary Thrombocytosis Patients
Source: Cancers (Basel). 2021 Jan 27;13(3):482. doi: 10.3390/cancers13030482 (PMC7865636; doi:10.3390/cancers13030482)
Supplement: Supplementary file 1 [file cancers-13-00482-s001.pdf]

cancers MDPI

Supplementary Materials: Polycythemia Vera and Essential Thrombocythemia patients exhibit unique serum metabolic profiles compared to healthy individuals and secondary thrombocytosis patients

Nuria Gómez-Cebrián, Ayelén Rojas-Benedicto, Arturo Albors-Vaquer, Carlos Besses, Beatriz Bellosillo, Joaquín Martínez-López, Antonio Pineda-Lucena and Leonor Puchades-Carrasco

Table S1. Clinical details available for the MPN patients included in the study.

Clinical Features	ET	PV
Hypertension, n (%)	17 (37%)	14 (63,7%)
High LDH levels, n (%)	3 (6.52%)	1 (4.54%)
Hemoglobin levels (g/dL; mean ± sd)	14.59 ± 1.31	17.47 ± 2.16
Leucocyte levels (x10^9/L; mean ± sd)	8.62 ± 2.09	9.45 ± 3.08
Platelets levels (x10^9/L; mean ± sd)	718.86 ± 277.92	504.3 ± 175.28
Treatment, n (%):		
• Hydroxyurea	29 (63.04%)	18 (81.81%)
• Acetylsalicylic acid	15 (32.61%)	4 (18.2%)
• Phlebotomy	2 (4.35%)	1 (4.54%)
• Interferon alpha	1 (2.17%)	-
• Any	13 (28.26%)	3 (13.63%)
Thrombotic events, n (%)		
• Before diagnosis	5 (10.87%)	5 (22.72%)
• At diagnosis	4 (8.67%)	1 (4.54%)
• After diagnosis	3 (6.52%)	2 (9.10%)
Other neoplasia, n (%)	5 (10.87%)	2 (9.10%)

ET: essential thrombocythemia, LDH: lactate dehydrogenase, n: number of patients, PV: polycythemia vera, sd: standard deviation.

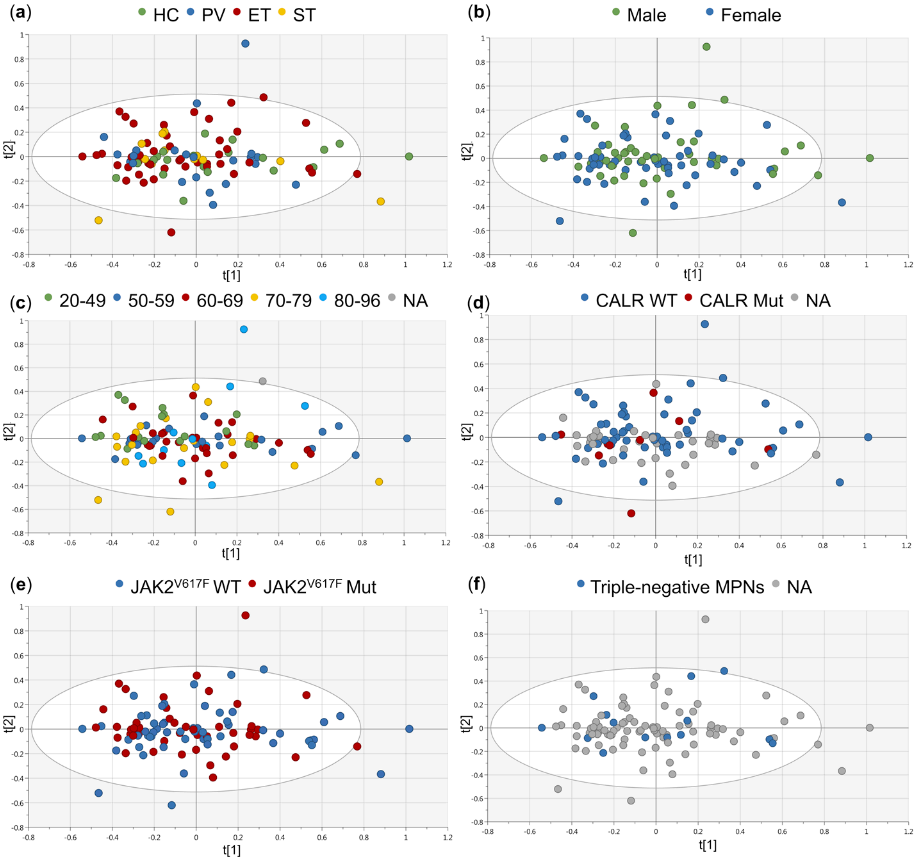

Figure S1. Principal Component Analysis (PCA) score plots corresponding to the samples included in the study. Samples are classified according to: (**a**) group of study, (**b**) gender, (**c**) age, (**d**) CALR, (**e**) JAK2V617F, and (f) mutational status. ET: essential thrombocythemia, HC: healthy controls, NA: not available, PV: polycythemia vera, ST: secondary thrombocythemia, Mut: Mutant, WT: Wild Type.

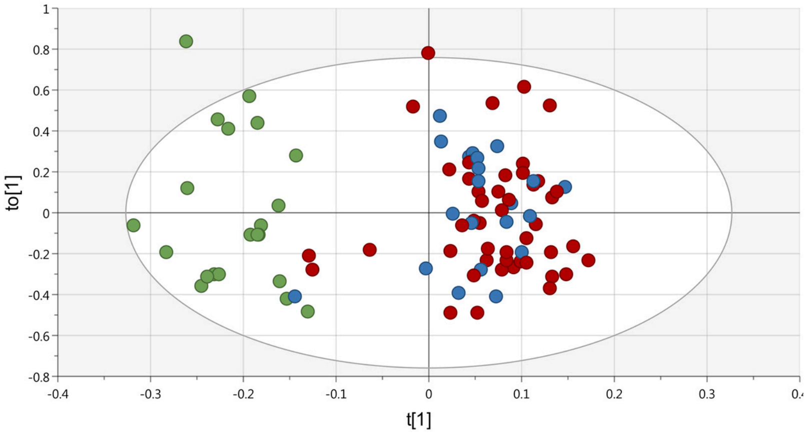

Figure S2. OPLS-DA scores plot for the comparison between HC (•) *vs* ET (•) + PV (•): R^2Y = 0.798, Q^2Y = 0.701. ET: essential thrombocythemia, HC: healthy controls, PV: polycythemia vera.

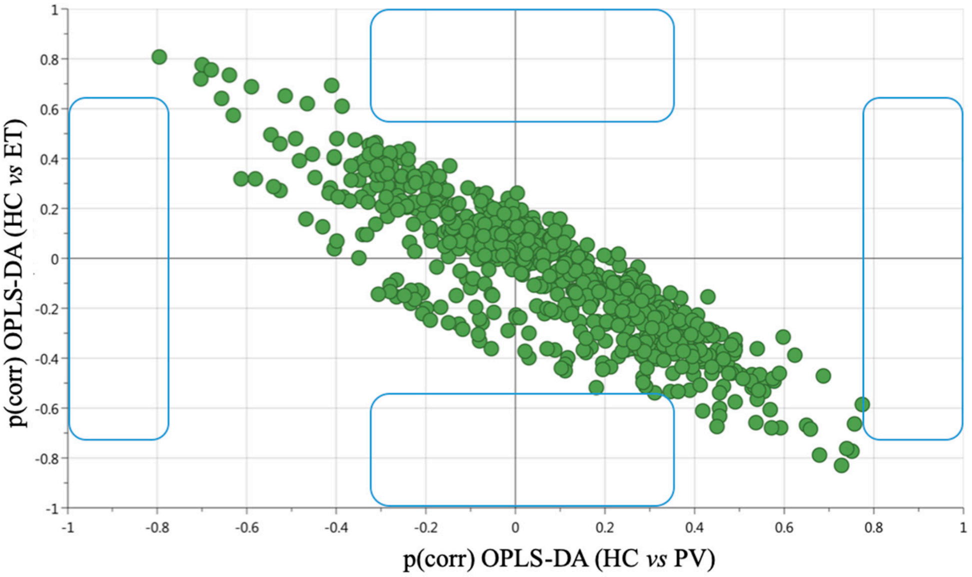

Figure S3. SUS-plot analysis correlating the OPLS-DA models of HC vs PV (x-axis) and HC vs ET (y-axis). Metabolic features that are equally important in both models are represented in the diagonal, while the blue rectangular frames represent unique metabolites in each model.
